# Supplementary material for: Ephrin-B1 regulates the adult diastolic function through a late postnatal maturation of cardiomyocyte surface crests
Source: eLife. 2023 Jan 17;12:e80904. doi: 10.7554/eLife.80904 (PMC9844986; doi:10.7554/eLife.80904)

Figure 1C: Original films (annotated, **A** or not, i,e, raw films **B**) from western-blot experiments to depict claudin-5 expression (dotted red lines) in the cardiac tissue during the postnatal maturation in rat, and corresponding GAPDH expression, that were cropped for illustration in Figure 1C in the original manuscript.
GAPDH was reprobed after stripping of the membrane following claudin-5 staining.
The membrane was cut in several parts to probe at the same time for different protein expression.

A

Claudin-5 blot

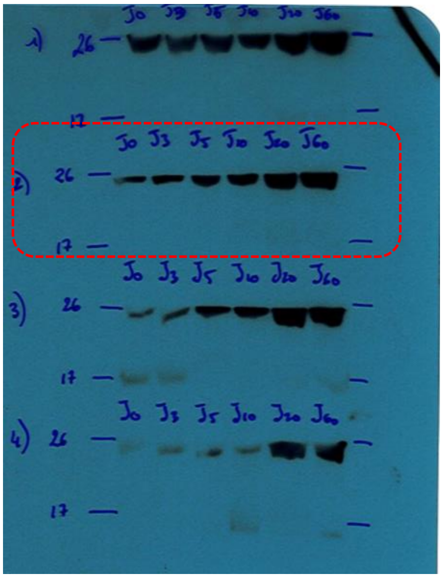

GAPDH blot

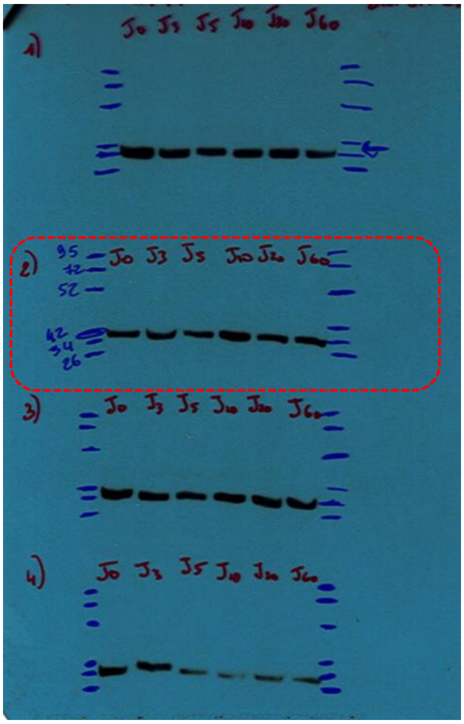

Numbers on the left indicate molecular weight markers (kDa)

B

Claudin-5 blot

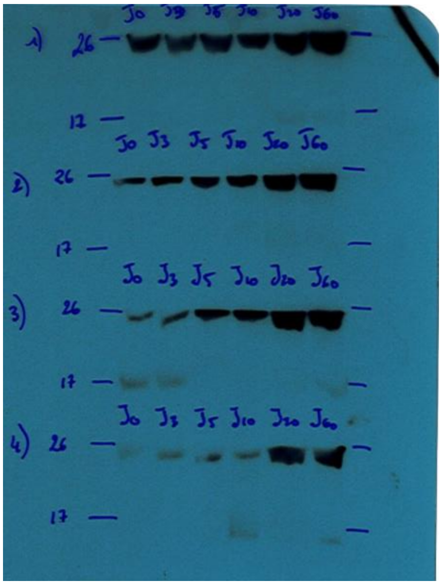

GAPDH blot

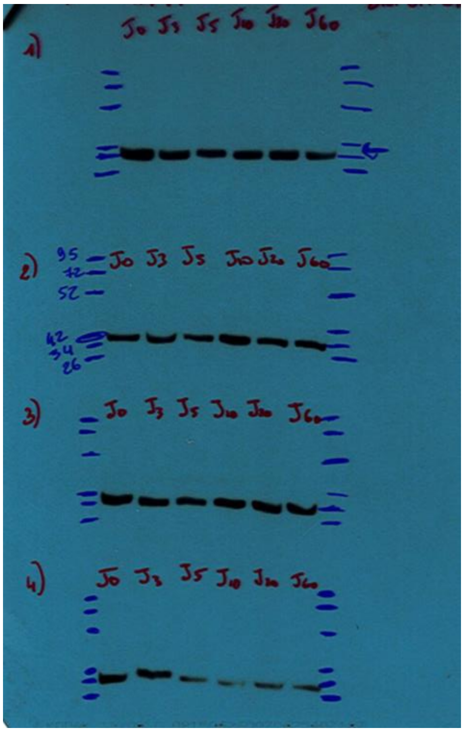

Supplement: Figure 1—source data 3. [file elife-80904-fig1-data3.pdf]
