# Supplementary material for: Ephrin-B1 regulates the adult diastolic function through a late postnatal maturation of cardiomyocyte surface crests
Source: eLife. 2023 Jan 17;12:e80904. doi: 10.7554/eLife.80904 (PMC9844986; doi:10.7554/eLife.80904)

Figure 5A: Original films (annotated, **A** or not, i,e, raw films **B**) from western-blot experiments to depict ephrin-B1 expression (dotted red lines) in the cardiac tissue during the postnatal maturation in rat, and corresponding GAPDH expression, that were cropped for illustration in Figure 5A in the original manuscript.
The membrane was cut in several parts to probe at the same time for different protein expression,

A

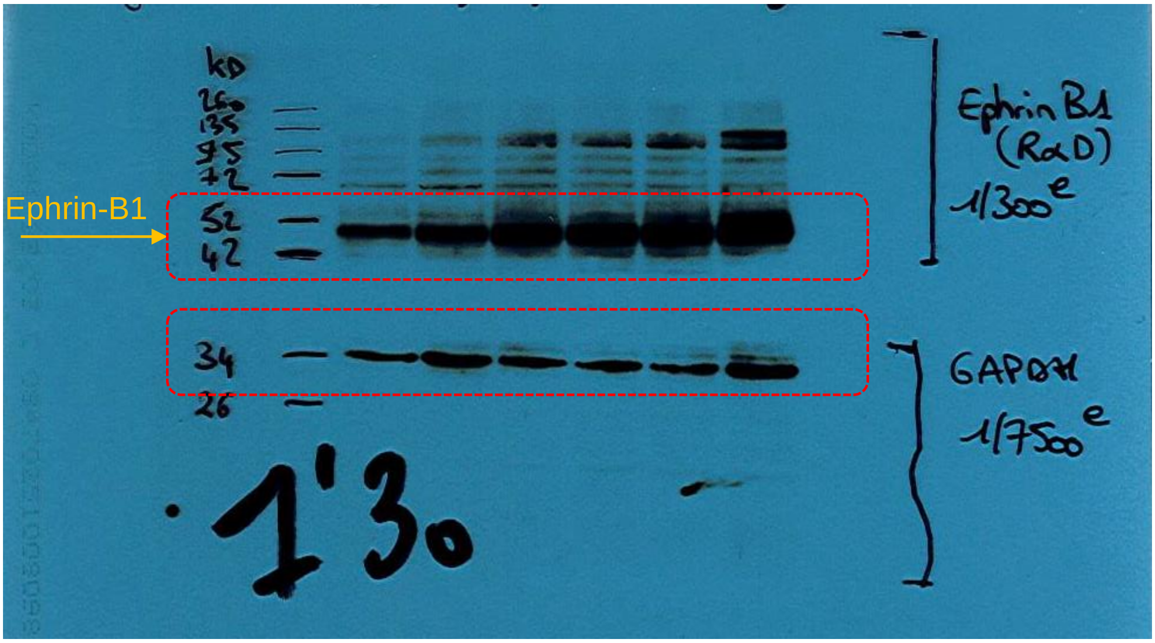

Numbers on the left indicate molecular weight markers (kDa)

B

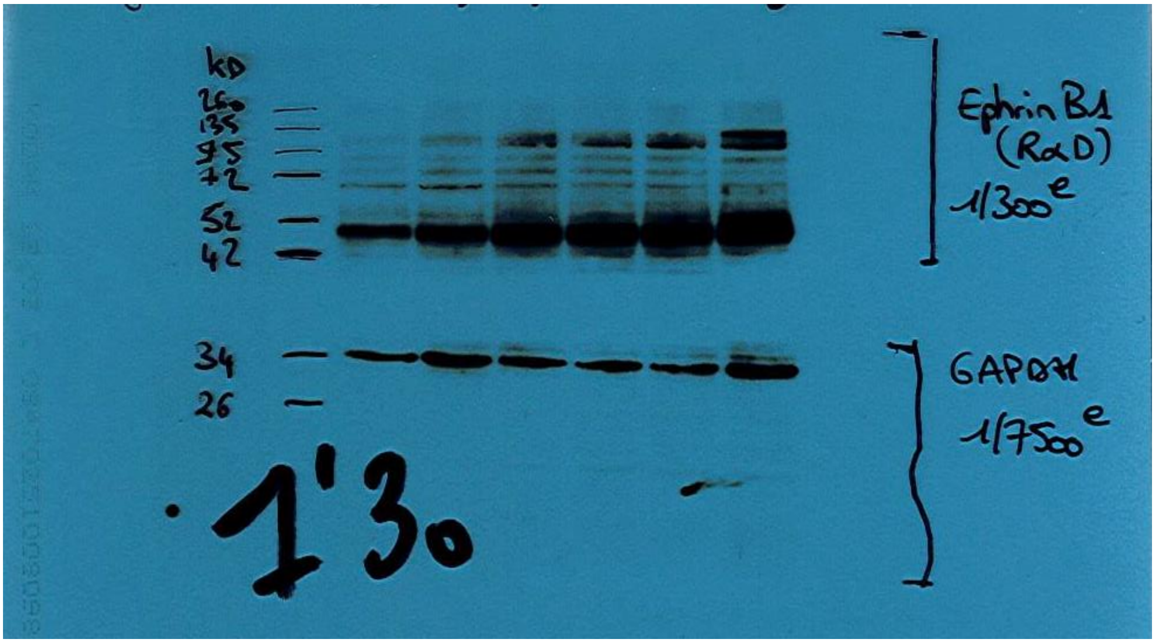

Supplement: Figure 5—source data 8. [file elife-80904-fig5-data8.pdf]
